# Transcriptional reprogramming of *Novacetimonas hansenii* SI1 during growth on glycerol

**DOI:** 10.1007/s00253-025-13583-2

**Published:** 2025-09-02

**Authors:** Małgorzata Wlaźlak, Izabela Cielecka, Maurycy Daroch

**Affiliations:** 1 Institute of Molecular and Industrial Biotechnology, Lodz University of Technology, B. Stefanowskiego 2/22, 90-537 Lodz, Poland; 2 School of Environment and Energy, Peking University Shenzhen Graduate School, Shenzhen, China

**Keywords:** *Novacetimonas*, Bacterial nanocellulose, Glycerol, RNA-seq, Transcriptomics

## Abstract

**Abstract:**

Bacterial nanocellulose (BNC) is a valuable biopolymer with immense potential in various sectors of biotechnology. However, large-scale production is hindered by low yields and high costs. Glycerol is an inexpensive and widely available carbon source for BNC biosynthesis, as it is a by-product of the biofuel industry. Compared to glucose, this polyol enhances BNC yields of *Novacetimonas hansenii* SI1 and related strains. This study investigates transcriptomic changes in *N. hansenii* SI1 after switching from glucose to glycerol using RNA-seq. The results reveal metabolic reprogramming, including upregulation of genes involved in glycerol uptake and catabolism, gluconeogenesis, the pentose phosphate pathway, and the Entner–Doudoroff pathway. Glycerol metabolism induces oxidative stress, evidenced by elevated expression of antioxidant enzymes, repair proteins, and metal ion homeostasis systems. Additionally, pathways such as riboflavin biosynthesis, methionine salvage, and sulphur assimilation are upregulated to mitigate oxidative damage. Increased oxidative conditions likely stimulate c-di-GMP synthesis, activating cellulose synthase and promoting BNC production. Furthermore, the acetan-like polymer biosynthetic pathway is significantly induced, further enhancing BNC yield. These findings expand our understanding of glycerol utilisation in BNC production, supporting cost-efficient and eco-friendly processes for maximising biopolymer exploitation.

**Key points:**

• *Growth on glycerol remodels central carbohydrate metabolism*

• *Glycerol metabolism induces oxidative stress*

• *Acetan-like biosynthesis and posttranslational effects stimulate BNC production*

**Supplementary Information:**

The online version contains supplementary material available at 10.1007/s00253-025-13583-2.

## Introduction

Bacterial nanocellulose (BNC) is a valuable polymer that continues to garner significant interest due to its unique properties. This linear polysaccharide, composed of D-glucopyranose units linked by β−1,4 glycosidic bonds, exhibits high purity, ultrafine fiber structure, high crystallinity, excellent mechanical strength, and remarkable water absorption capacity, along with biocompatibility. Its applications span diverse biotechnological sectors, including prominent fields such as the food and cosmetics industries, medicine, and advanced material technologies (Cazón and Vázquez 2021; Stasiak-Różańska et al. 2024; Kaczmarek and Białkowska 2025; Venturelli et al. 2025). The most efficient BNC producers belong to acetic acid bacterial strains within the *Komagataeibacter* and *Novacetimonas* genera (Ryngajłło et al. 2020).

The application and industrial-scale production of BNC are constrained by low production rates. To address this challenge, several strategies have been deployed (Ryngajłło et al. 2020; Potočnik et al. 2023; Pedroso-Roussado 2024). These primarily include optimising bioprocess conditions (Cielecka et al. 2020, 2021a; Rackov et al. 2025), isolating highly efficient bacterial strains (Gullo et al. 2017; Anguluri et al. 2022; Karničnik et al. 2025), or genetically engineering producer strains to enhance BNC synthesis (Florea et al. 2016; Montenegro-Silva et al. 2024; Yang et al. 2024; Lasagni et al. 2024; Huang et al. 2025).

An important factor that primarily determines the overall economic efficiency of a bioprocess is the growth medium price, which is estimated to account for 50–65% of total production costs (Hussain et al. 2019). Therefore, developing cost-effective culture media for BNC biosynthesis represents a critical strategy to reduce production expenses. Of particular interest is the utilisation of low-cost alternative carbon sources or by-products and waste materials from agricultural, food, or biofuel industries (Moon et al. 2006; Wu and Liu 2013; Brugnoli et al. 2023).

Glycerol (1,2,3-propanetriol), a widely available and affordable substrate, can be efficiently used in fermentation processes. Large quantities of glycerol are generated as a by-product from biodiesel and other oleochemical industries, which apply technologies involving triglyceride transesterification reactions. Biodiesel-derived glycerol is predominantly marketed in unrefined, crude form, which contains impurities (residual catalyst, water, methanol, fatty acids, soaps, and inorganic salts). Despite its impurities, studies have demonstrated that unrefined glycerol can be fermented by various microorganisms to produce a wide range of bioproducts (Wang et al. 2024). The affordability of crude glycerol stems from its oversupply and limited market demand, driven by the high cost of purification and the low market capacity to absorb its ever-increasing production (Moklis et al. 2024; Tsouko et al. 2024).

Glycerol naturally occurs in environments inhabited by *Komagataeibacter* and *Novacetimonas* species such as wines, spirits, kombucha, and other fermented beverages, since it is the major by-product of ethanol fermentation by yeast (Scanes et al. 1998; Zhao et al. 2015; Dartora et al. 2023). These bacteria were shown to efficiently synthesise BNC on media containing glycerol as the main carbon source, both in its pure and unrefined form (Kim et al. 2006; Zhong et al. 2013; Tsouko et al. 2015, 2024; Thorat and Dastager 2018; Ryngajłło et al. 2019b; Gupte et al. 2021; Zikmanis et al. 2021).

Glycerol catabolism has been most extensively studied in *Escherichia coli* and *Pseudomonas* species (Cozzarelli et al. 1968; Zeng et al. 1996; Martínez-Gómez et al. 2012; Nikel et al. 2014; Poblete-Castro et al. 2020). In these species, as well as in most Gram-negative bacteria, glycerol metabolism typically proceeds through two alternative pathways. The first involves ATP-dependent phosphorylation of glycerol, while the second entails direct oxidation of the polyol into dihydroxyacetone (DHA). Based on genomic analyses and biochemical studies, it is evident that metabolic networks of BNC producers within the *Komagataeibacter* and *Novacetimonas* genera contain the necessary enzymatic components required for glycerol catabolism (Weinhouse and Benziman 1976; Nabe et al. 1979; Zhong et al. 2013; Mamlouk and Gullo 2013; Ryngajłło et al. 2019b).

The first pathway of glycerol assimilation is mediated by the proteins of the *glp* regulon (Poblete-Castro et al. 2020). In *Komagataeibacter* and *Novacetimonas*, this regulon consists of six genes (*glpF*, *glpD*, *glpK*, *glpX*, *fba*, *glpR*) clustered together (Ryngajłło et al. 2019b). Glycerol enters the cell via passive or facilitated diffusion. Uptake of glycerol, especially at low concentrations, is facilitated by the membrane-bound GlpF protein. Inside the cell, glycerol is phosphorylated to *sn*-glycerol-3-phosphate (G3P) by glycerol kinase (GlpK). Subsequently, aerobic G3P dehydrogenase (GlpD) oxidises G3P to dihydroxyacetone phosphate (DHAP). DHAP may enter glycolysis via glyceraldehyde-3-P or follow the gluconeogenesis pathway by the action of fructose-bisphosphate aldolase (Fba) and then be converted to fructose-6-phosphate by fructose-1,6-bisphosphatase (GlpX). It has been shown in *E. coli* that the *glp* operon is regulated by GlpR, a DeoR family transcriptional repressor that releases repression in the presence of G3P (Zeng et al. 1996).

Another route of glycerol dissimilation in the discussed BNC producers involves direct oxidation of glycerol to DHA in the periplasm by the pyrroloquinoline quinone (PQQ)-dependent glycerol dehydrogenase (SldAB; (Adachi and Yakushi 2016). It has been shown in *K. xylinus* (*Acetobacter xylinum* in the original work) that the same enzyme is responsible for further ATP-dependent phosphorylation of both glycerol and DHA (Weinhouse and Benziman 1976).

In our previous work involving *N. hansenii* SI1, we observed that glycerol was the preferred carbon source, resulting in the highest BNC yield compared to tested sugars, including glucose (Cielecka et al. 2021b). In this study, we aimed to elucidate the molecular mechanism underlying enhanced BNC synthesis on glycerol. To this end, we sequenced mRNA extracted from *N. hansenii* SI1 cells grown on glycerol and compared global gene expression with a reference medium containing glucose as the main carbon source. We analyzed transcriptomic changes in central metabolism and pathways related to exopolysaccharides (EPS) biosynthesis. Notably, we uncovered transcriptional reprogramming of the pathways not previously associated with EPS biosynthesis in this and other related strains.

This research contributes a relevant perspective towards the efficient use of glycerol as a substrate for BNC biosynthesis, which is important for improving the economy of this bioprocess.

## Material and methods

### Strain growth, isolation, and sequencing of RNA

In the case of glucose cultures, the *N. hansenii* SI1 strain was cultured in SH medium (glucose medium; Hestrin and Schramm 1954) as described in Cielecka et al. (2021b). In the case of glycerol cultures, the culture conditions were the same. Bacterial cells from a glycerol stock were streaked onto the SH agar plates and incubated for 5 days at 30 °C. Next, 5 mL of SH medium in 10 mL test tubes was inoculated with a single colony taken from the plate and cultured at 30 °C under static conditions for 2 days. One litre of the culture medium contained 20.0 g of glycerol (Chempur, Piekary Śląskie, Poland), 5.0 g yeast extract (BTL, Łódź, Poland), 5.0 g bacterial peptone (BTL, Łódź, Poland), 2.7 g sodium phosphate dibasic (Chempur, Piekary Śląskie, Poland), 1.15 g citric acid (Chempur, Piekary Śląskie, Poland), and 0.5 g magnesium sulphate (Chempur, Piekary Śląskie, Poland). The initial pH of the medium was adjusted to 5.7 with 80% acetic acid (Chempur, Piekary Śląskie, Poland). The cultures were prepared in triplicate. The *N. hansenii* SI1 strain can be obtained from the corresponding author upon request.

The culture was harvested on the second day, which corresponds to the end of the exponential growth phase. At the end of the culture, 1% of cellulase solution (v/v; from *Trichoderma reesei* ATCC 26921, Sigma-Aldrich, Steinheim, Germany) was added to each of the cultures, which were next incubated for 3 h at 30 °C under static conditions with intermittent vortexing. Afterwards, total RNA was isolated from 4 mL of the incubated mixture using the GeneMATRIX Universal RNA Purification Kit (EURX, Gdańsk, Poland). Three RNA samples were prepared from separate cultures.

The isolated RNA was next subjected to high-throughput sequencing. First, NGS libraries were prepared using the RNA Library Prep Set (MGI, Shenzhen, China), incorporating a ribodepletion procedure. For the glycerol cultures, 33,782,312 pairs of 100 bp reads were generated by the MGISEQ-2000 sequencer (MGI, Shenzhen, China). NGS library preparation and sequencing were performed at the BGI-TECH (Wuhan, China). The raw reads were deposited in the NCBI database under the BioProject accession: PRJNA821681 and BioSample accessions: SAMN42361398–SAMN42361400.

For the control condition cultures (glucose medium), we re-used the libraries deposited in our previous study (BioProject accession: PRJNA821681 and BioSample accessions: SAMN37700948–SAMN37700950), which consisted of 30,174,694 pairs of 100 bp reads in total. All libraries analyzed in this study were generated from the same experimental series.

### Differential gene expression analysis

The sequenced reads were mapped to the *N. hansenii* SI1 genome (GenBank accession number: GCA_037913045.1) using Bowtie2 (v. 2.3.5.1; Langmead and Salzberg 2012), and the SAM files were compressed to BAM files using samtools (v. 1.9; Li et al. 2009). The mapped reads were further processed using a custom pipeline in R (v. 3.5.1) employing Rsamtools (v. 1.34.0; Morgan and Pagès 2017), GenomicAlignments (v. 1.18.0; Lawrence et al. 2013), and GenomicFeatures (v. 1.34.1; Lawrence et al. 2013) Bioconductor packages (Gentleman et al. 2004; Huber et al. 2015). The same genome annotation, from Prokka and RAST, as previously described was used in this study (Ryngajłło et al. 2024). Only the reads that mapped uniquely to the annotated CDSs were submitted to further analyses.

Differential gene expression analysis (DGEA) was conducted using DESeq2 (v. 1.22.2; Love et al. 2014). Fragments per kilobase per million mapped fragments (FPKM) were calculated using DESeq2. The results of DGEA are presented in Online Resource 2: Table S1. Significantly differentially expressed genes (DEGs) were filtered using a threshold for the adjusted *p-*value < 0.01 and |log_2_FC|≥1 (fold change) cut-off.

Changes in gene expression (log_2_fold) were visualised in the context of metabolic pathways, which were drawn and annotated using PathVisio (v. 3.3.0; van Iersel et al. 2008; Kutmon et al. 2015). Functional enrichment for DEGs was performed on RAST annotation, where 1294 genes (42%) were annotated with at least one RAST category. Multi-category proteins (assigned to more than one RAST category) were counted in each of their categories. Functional enrichment was conducted using Fisher’s exact test (one-tailed) as implemented in R. The false discovery rate (FDR) was controlled using the Benjamini–Hochberg procedure (Benjamini and Hochberg 1995). The results are presented in Online Resource 2: Table S2. Processing of annotation information was done using custom-made scripts written in Perl v. 5.26.2 or Python v. 3.7.9.

The calculations mentioned in this paper were performed using the BlueOcean computational cluster, which is part of the TUL Computing & Information Services Centre infrastructure.

### Prophage region prediction

Regions encoding prophage proteins in the *N. hansenii* SI1 genome were predicted using the PHASTEST web server (access date: September 2024; Zhou et al. 2011; Arndt et al. 2016; Wishart et al. 2023). Sequence coverage for the tested samples was visualised using IGV (v. 2.3.91; Thorvaldsdóttir et al. 2013). Read counts from each RNA-seq library were normalised using RPKM and transformed to bigWig format using the bamCoverage module of the deepTools (v. 2.0) Python package (Ramírez et al. 2016).

### Other bioinformatic analyses and predictions

Transmembrane proteins were predicted using the Phobius web server (access date: September 2024; Käll et al. 2007).

An additional search for homologous proteins discussed in this study was performed using NCBI blastp (v. 2.12.0 +; Altschul et al. 1990; Camacho et al. 2009) by applying the threshold of 0.005 for *E*-value, 30% sequence identity, and 70% query sequence coverage. The *N. hansenii* SI1 proteome predicted by Prokka was used. The amino acid sequences of the query proteins were downloaded from the UniProt database (Release 2024_04).

## Results

### Differential gene expression analysis and functional enrichment of DEGs

**Fig. 1:**
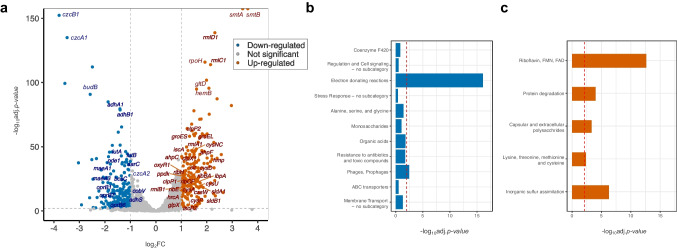
Analysis of gene expression and functional characterisation of DEGs between the glycerol and the glucose cultures of *N. hansenii* SI1. **a** Volcano plot representing gene expression changes. Shown are − log_10_ of adjusted *p-values* versus log_2_ fold changes (FC). The repressed and the induced DEGs are colored in blue and orange, respectively. Gene symbols are plotted only for the DEGs. The dashed horizontal line indicates the adjusted *p-value* threshold of 0.01. The dashed vertical lines indicate the |log_2_FC|= 1 thresholds. **b**, **c** Results of functional enrichment analysis of DEGs based on RAST annotation. Displayed are enriched RAST subcategories. The enriched functional bins of the downregulated and the upregulated DEGs are displayed as blue and orange bars, respectively. Shown are the results of the one-sided Fisher’s exact test (one-tailed). For clarity, only the bins that scored the adjusted *p-value* below 0.5 are presented. The red dashed vertical line indicates the adjusted *p-value* threshold of 0.01

To understand the molecular changes in *N. hansenii* SI1 associated with growth on glycerol as the main carbon and energy source, we performed comparative transcriptomic analysis. Gene expression analysis identified 424 significantly differentially expressed genes (13% of total), with 219 downregulated and 205 upregulated (Fig. 1a). Initial screening revealed few annotated genes in this list; subsequent RAST annotation-based analysis provided deeper insights into functional assignments.

The results of the gene set enrichment analysis yielded insights into functions overrepresented among the DEGs. Only three RAST functional categories demonstrated significant enrichment across both the downregulated and upregulated genes. These categories, ordered by ascending adjusted *p-value*, were “respiration” and “phages, prophages, transposable elements, plasmids” for the downregulated DEGs (Online Resource 1: Fig. S1a) and “sulphur metabolism” in the case of the upregulated DEGs (Online Resource 1: Fig. S1b). More detailed functions were exposed at the subcategory level (Fig. 1b, c). The significantly overrepresented RAST subcategories among the repressed DEGs included “electron donating reactions” and “phages, prophages” (Fig. 1b). For the induced DEGs, these were “riboflavin, FMN, FAD”, “inorganic sulphur assimilation”, “protein degradation”, “capsular and extracellular polysaccharides”, and “lysine, threonine, methionine, and cysteine” (Fig. 1c). Finally, at the subsystem level, several functions were enriched, and among them, significantly enriched subsystems included “NADH ubiquinone oxidoreductase”, “respiratory complex I”, and “cobalt–zinc–cadmium resistance” for the downregulated DEGs. For the upregulated DEGs, these were “inorganic sulphur assimilation”, “cysteine biosynthesis”, “riboflavin, FMN and FAD metabolism”, “riboflavin, FMN and FAD metabolism in plants”, “riboflavin to FAD”, “proteasome bacterial”, “dTDP-rhamnose synthesis”, and “rhamnose containing glycans”. These results guided further, detailed analysis of the enriched functional subcategories.

### Gene expression changes in the central carbohydrate metabolism pathways

**Fig. 2:**
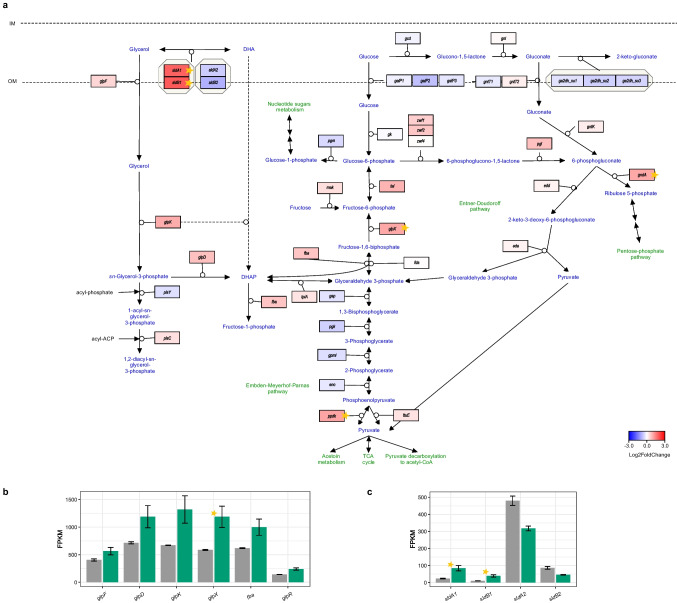
Transcriptomic changes in the central carbohydrate metabolism pathway between *N. hansenii* SI1 cultures grown in the glycerol and the glucose medium. **a** The pathway of glycerol metabolism and glycolysis/gluconeogenesis. Genes are colored according to log_2_ fold change in expression between the glycerol and the glucose cultures. IM, inner membrane; OM, outer membrane. **b** Expression of the glycerol operon (*glp*). **c** Glycerol dehydrogenase subunits of two loci. Transcripts mean FPKM values are shown in either gray or green for cells grown in the glucose or the glycerol medium, respectively. Bars represent the means from 3 replicated cultures. Thin black bars denote standard error. Stars in all panels denote statistically significant changes (called by DESeq2; adjusted *p-value* < 0.01 and |log_2_FC|  ≥ 1)

During growth on glycerol, oxidation of glucose in the periplasm, along with its uptake and phosphorylation in the cytosol, exhibited mild repression (Fig. 2). The Embden–Meyerhof–Parnas pathway was downregulated. Conversely, both the Entner–Doudoroff (ED) pathway and the pentose phosphate pathway (PPP) were induced, with significant changes observed in the gene encoding 6-phosphogluconate dehydrogenase (*gndA*, log_2_FC = 1.0, adj. *p-value* = 5.22 * 10^−18^; Fig. 2 and Online Resource 1: Fig. S2).

The pathway for glycerol uptake and catabolism was upregulated (Fig. 2a), with strong induction observed in genes within the *glp* cluster (Fig. 2b). The genes on the pathway from DHAP to glucose-6-phosphate, such as *tpiA*, *fba*, *tal*, and *glpX,* were upregulated, where, for the last one, this change was significant (log_2_FC = 1.0, adj. *p-value* = 1.23 * 10^−8^; Fig. 2a).

The genes encoding copies of large and small subunits of glycerol dehydrogenase are located in two loci in the genome of *N. hansenii* SI1 (*sldAB1*, *sldAB2*). The *sldAB*2, of which expression is the highest of the two gene sets, was mildly downregulated in the glycerol medium (Fig. 2a and 2c). The expression of *sldAB1*, which is lower than that of *sldAB2*, is significantly induced in the glycerol medium (log_2_FC = 1.9 for both subunits, adj. *p-value* = 1.23 * 10^−8^ (*sldA1*) and 4.62 * 10^−8^ (*sldB1*)).

The *sldAB1* genes are located in close proximity to a gene cluster which encloses predicted tagatose kinase, tagatose 6-phosphate 4-epimerase, sorbitol dehydrogenase, and putative metabolite transport protein (Online Resource 1: Fig. S3a). These genes are all significantly upregulated (Online Resource 1: Fig. S3b).

The metabolism of DHA produced by glycerol dehydrogenase is not completely clear. Based on the functional predictions, the genome of *N. hansenii* SI1 does not encode dihydroxyacetone kinase, as is the case with the strains of the *Komagataeibacter* genus and *Novacetimonas maltaceti* LMG 1529 (based on UniProt database search, data not shown). One possibility is that phosphorylation of DHA is conducted by glycerol kinase (*glpK*), since this activity was found for its ortholog in *K. xylinus* (*Acetobacter xylinum* in the original work (Weinhouse and Benziman 1976)). This gene appears to be the sole candidate for this function in the *N. hansenii* SI1 genome.

### Gene expression changes in the TCA cycle, acetoin metabolism, and respiration

**Fig. 3:**
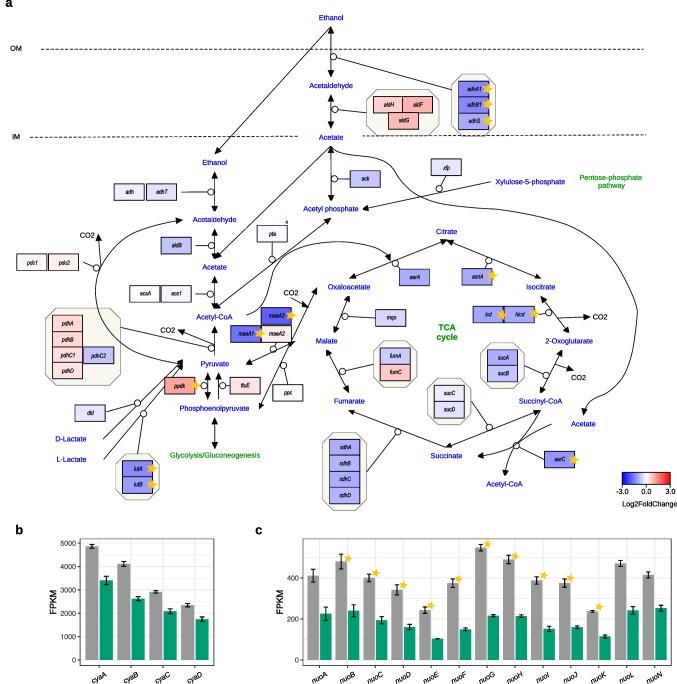
Changes in the expression of genes involved in the TCA cycle and respiration in *N. hansenii* SI1. **a** Transcriptomic changes in the TCA cycle. Genes are colored according to log_2_ fold change in expression between glycerol and glucose cultures. Stars denote statistically significant changes (called by DESeq2; adjusted *p-value* < 0.01 and |log_2_FC| ≥ 1). IM, inner membrane; OM, outer membrane. **b** Mean expression levels of the subunits of cytochrome ba(3) ubiquinol oxidase (UOX). **c** Mean expression levels of the subunits of NADH-quinone oxidoreductase operon (*nuo*, complex I). Transcripts’ mean FPKM values are shown either gray or green for cells grown in either the glucose or glycerol medium, respectively. Bars represent the means from 3 replicated cultures. Thin black bars denote standard error. Stars in all panels denote statistically significant changes (called by DESeq2; adjusted *p-value* < 0.01 and |log_2_FC|  ≥ 1)

The tricarboxylic acid (TCA) cycle was overall downregulated (Fig. 3a), which agreed with the results of RAST enrichment analysis (Fig. 1b). Many genes of this pathway were significantly repressed. These were such genes as *maeA1* (log_2_FC =  − 1.7, adj. *p-value* = 1.44 * 10^−43^), *maeA3* (log_2_FC =  − 1.9, adj. *p-value* = 9.15 * 10^−23^), *acnA* (log_2_FC =  − 1.0, adj. *p-value* = 3 * 10^−86^), *icd* (log_2_FC =  − 1.1, adj. *p-value* = 6.48 * 10^−37^), *hicd* (log_2_FC =  − 1.0, adj. *p-value* = 2.4 * 10^−28^), *aarC* (log_2_FC =  − 1.22, adj. *p-value* = 5.72 * 10^−36^).

Other genes associated with the TCA cycle, such as those encoding L-lactate dehydrogenase and alcohol dehydrogenase subunits, were significantly repressed (*lutA* (log_2_FC =  − 1.4, adj. *p-value* = 1.44 * 10^−43^), *lutB* (log_2_FC =  − 1.2, adj. *p-value* = 8.55 * 10^−41^), *adhA1* (log_2_FC =  − 1.4, adj. *p-value* = 3.02 * 10^−80^), *adhB1* (log_2_FC =  − 1.4, adj. *p-value* = 2.21 * 10^−79^), *adhS* (log_2_FC =  − 1.0, adj. *p-value* = 4.44 * 10^−13^); Fig. 3a). In contrast, the genes encoding pyruvate phosphate dikinase, the subunits of pyruvate dehydrogenase, and aldehyde dehydrogenase were induced, where for *ppdk* this change was significant ((log_2_FC = 1.1, adj. *p-value* = 1.17*10^−27^); Fig. 3a).

Additionally, we focused on another pathway associated with the TCA cycle and connected to acetoin metabolism. The two genes of this operon, *budB* and *alsD*, are significantly repressed in the glycerol medium (log_2_FC =  − 3.1, adj. *p-value* = 9.82*10^−231^ and log_2_FC =  − 2.6, adj. *p-value* = 1.53*10^−91^, respectively). The products of this operon, acetolactate synthase (*budB*) and alpha-acetolactate decarboxylase (*alsD*), are involved in acetoin biosynthesis. Our transcriptomic results show that the putative pathway of acetoin metabolism is generally downregulated when the cells are grown on the glycerol medium (Online Resource 1: Fig. S4).

Finally, we investigated the response of genes associated with the respiratory apparatus. Oxidation of substrates in the periplasm by membrane-bound dehydrogenases is coupled to the reduction of ubiquinone to ubiquinol. Our results indicate mild downregulation of the genes encoding the subunits of the ba_3_-type ubiquinol oxidase (Fig. 3b). Cytoplasmic enzymes assimilate these oxidation products and further metabolise substrates to generate NADH. The expression of the *nuo* operon of the NADH dehydrogenase (complex I) was strongly repressed, with most genes showing significant expression changes (Fig. 3c).

The results obtained at this stage have shown that, when the *N. hansenii* SI1 cells are grown on the glycerol medium, TCA cycle progression, organic acid production, and cytoplasmic (NADH) respiration are repressed.

### Transcriptional activation of riboflavin biosynthesis pathway

**Fig. 4:**
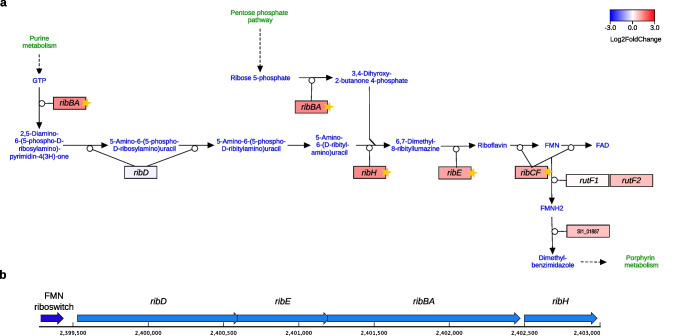
The predicted riboflavin biosynthesis pathway in *N. hansenii* SI1. **a** Transcriptomic changes in the riboflavin biosynthesis pathway (based on Solopova et al. 2020). Genes are colored according to log_2_ fold change in expression between glycerol and glucose cultures. Stars denote statistically significant changes (called by DESeq2; adjusted *p-value* < 0.01 and |log_2_FC| ≥ 1). **b** Gene loci encoding the predicted FMN riboswitch and the riboflavin synthesis enzymes in the genome of *N. hansenii* SI1. The gene identifiers and symbols correspond to the following predicted functions: *ribD—*bifunctional diaminohydroxyphosphoribosylaminopyrimidine deaminase (EC 3.5.4.26)/5-amino-6-(5-phosphoribosylamino)uracil reductase (EC 1.1.1.193); *ribH—*6,7-dimethyl-8-ribityllumazine synthase (EC 2.5.1.78); *ribE—*riboflavin synthase (EC 2.5.1.9); *ribBA—*bifunctional GTP cyclohydrolase II (EC 3.5.4.25); 3,4-dihydroxy-2-butanone 4-phosphate synthase (EC 4.1.99.12); *ribCF—*bifunctional riboflavin kinase (EC 2.7.1.26)/FAD synthase (EC 2.7.7.2); *rutF—*FMN reductase; SI1_01887—5,6-dimethylbenzimidazole synthase

In agreement with the results of RAST enrichment analysis, we found that the riboflavin biosynthesis pathway was upregulated, with the majority of genes in this pathway showing significant expression changes (Fig. 4a). The highest expression changes were displayed by the *rib* genes (log_2_FC > 1 and adj. *p-value* < 0.01 for most of these genes), of which *ribD*, *ribC*, *ribBA*, and *ribE* likely form a transcriptional unit (Fig. 4b). A predicted FMN riboswitch is located upstream of the *rib* cluster, which may regulate FMN/FAD biosynthesis (Fig. 4b).

The *ribCF* gene is located in a different genome location than the *rib* cluster and forms a putative cluster with genes (*mntC*, *mntD*, and *mntB*) that are predicted to be involved in the methionine salvage pathway (Online Resource 1: Fig. S5a). These genes, similarly to *ribCF,* are significantly upregulated in the glycerol medium (Online Resource 1: Fig. S5b). Based on the genome annotation, other genes of this pathway are mildly upregulated (*mtnK* and *mtnA*) or downregulated (*mtnP* and *mtnN*).

### Expression of sulphur assimilation and cysteine biosynthesis pathway

**Fig. 5:**
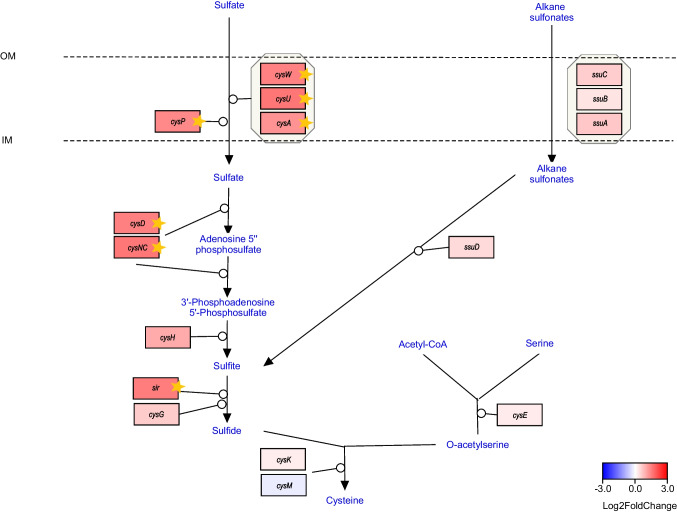
Transcriptomic changes in the predicted pathway of sulphur assimilation and cysteine biosynthesis in *N. hansenii* SI1. Genes are colored according to log_2_ fold change in expression between glycerol and glucose cultures. Stars denote statistically significant changes (called by DESeq2; adjusted *p-value* < 0.01 and |log_2_FC| ≥ 1)

One of the significantly enriched RAST functional subcategories was “inorganic sulphur assimilation” (Fig. 1c). Upon examining the sulphur assimilation pathway, we observed its general activation in the culture grown on the glycerol medium (Fig. 5). Specifically, genes involved in sulphate transport and its reduction to sulphide were predominantly upregulated.

The genes associated with alkanesulfonate transport and metabolism were mildly induced, while the downstream portion of the cysteine biosynthesis pathway did not exhibit significant changes in expression (Fig. 5).

### Protein degradation and stress response

**Fig. 6:**
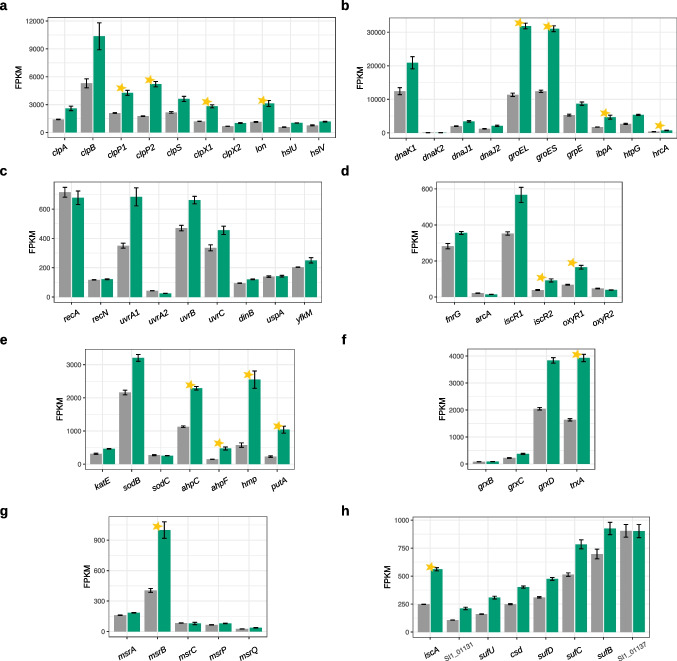
Expression changes in genes associated with protein degradation and stress response. **a** ATP-dependent proteases. **b** Molecular chaperones. **c** DNA damage and the general stress-responsive genes. **d** Transcription regulators associated with oxidative stress response. **e** Genes coding for oxidative stress-responsive enzymes. **f** Glutaredoxins (*grx*) and thioredoxin A (*trxA*) genes. **g** Genes encoding methionine sulphoxide reductases (*msr*). **h** Suf cluster of genes involved in Fe–S cluster repair. Transcript mean FPKM values are shown in gray or green for cells grown in either the glucose or the glycerol medium, respectively. Bars represent the means from 3 replicated cultures. Thin black bars denote standard error. Stars in all panels denote statistically significant changes (called by DESeq2; adjusted *p-value* < 0.01 and |log_2_FC|  ≥ 1)

The results of functional enrichment analysis revealed particular induction of the genes involved in protein degradation (Fig. 1c). Indeed, we observed that the genes encoding ATP-dependent proteases were mostly upregulated, and some of them (*clpP1*, *clpP2*, *clpX1*, *lon*) significantly (Fig. 6a). A comparable pattern was observed for the majority of genes encoding molecular chaperones (Fig. 6b). Here, such genes as *groEL*, *groES*, *ibpA*, and *hrcA* were significantly induced.

Further analysis revealed mild upregulation of genes linked to DNA damage and the general stress response (Fig. 6c).

The expression of homologs of transcription factors associated with the oxidative stress response was mostly induced in the culture grown on the glycerol medium (Fig. 6d). For *iscR*2 and *oxyR1*, this change was significant (log_2_FC = 1.25, adj. *p-value* = 1.29 * 10^−13^ and log_2_FC = 1.28, adj. *p-value* = 2.59 * 10^−28^, respectively). More severe upregulation was displayed by the genes encoding enzymes involved in the oxidative stress response (Fig. 6e). Here, such genes as *ahpC*, *ahpF*, *hmp*, and *putA* were significantly induced, where for *hmp* and *putA* the log fold change was greater than 2 (log_2_FC = 2.15, adj. *p-value* = 4.70 * 10^−39^ for *hmp* and log_2_FC = 2.19, adj. *p-value* = 1.64 * 10^−43^ for *putA*).

Beyond antioxidant enzymes, genes encoding repair proteins for oxidised molecules were induced. One such group were the genes coding for three predicted glutaredoxins (*grx*) and thioredoxin A (*trxA*), where for the *trxA* gene the change in expression was significant (log_2_FC = 1.26, adj. *p-value* = 8.09*10^−48^; (Fig. 6f)).

Methionine sulphoxide reductases (*msr*), which reduce free and protein-based methionine sulphoxides to methionine, were also upregulated. Among the five predicted *msr* genes in the *N. hansenii* SI1 genome (*msrA*, *msrB*, *msrC*, *msrP*, and *msrQ*), only *msrB* exhibited significant upregulation (log_2_FC = 1.30, adj. *p-value* = 1.01 * 10^−24^; Fig. 6g).

Additionally, the *suf* cluster genes, involved in Fe–S cluster repair, displayed mild induction (Fig. 6h). Among its eight genes, only *iscA*, which is located upstream of the *suf* cluster, showed significant changes (log_2_FC = 1.18, adj. *p-value* = 2.79*10^−45^).

### Metal homeostasis

Guided by the results of functional enrichment of RAST subcategories, we explored first the “cobalt-zinc-cadmium resistance” subsystems, both of which were significantly enriched among the downregulated DEGs (Fig. 1b and Online Resource 1: Fig. S1c).

**Fig. 7:**
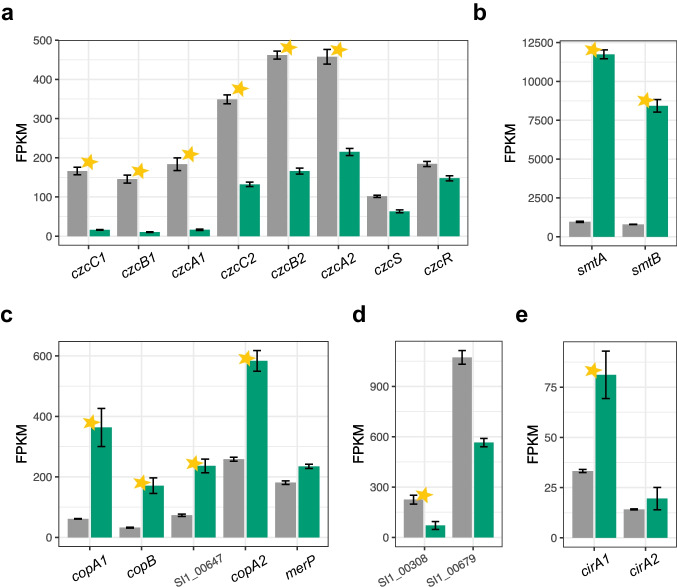
Expression changes in genes encoding proteins involved in metal homeostasis. **a** Two gene clusters (*czc1* and *czc2*) encoding homologs of the cobalt-zinc-cadmium resistance system. **b** Genes encoding homologs of SmtA and SmtB proteins involved in zinc and other essential ions homeostasis. **c** Copper resistance genes (*copA*1, *copB*, SI1_00647 [unknown function], and *copA*2) and a predicted *merP* gene encoding a mercury scavenger. **d** Genes encoding the most highly expressed TonB-dependent receptors. **e** Genes coding for homologs of the colicin I receptor, CirA. Transcript mean FPKM values are shown in gray or green for cells grown in either the glucose or glycerol medium, respectively. Bars represent the means from 3 replicated cultures. Thin black bars denote standard error. Stars in all panels denote statistically significant changes (called by DESeq2; adjusted *p-value* < 0.01 and |log_2_FC| ≥ 1)

Based on the *N. hansenii* SI1 genome annotation, two gene clusters (*czcCBA1* and *czcCBA2*) encoding homologs of the cobalt-zinc-cadmium resistance system CzcCBA of *Cupriavidus metallidurans* CH34 (Janssen et al. 2010) were identified. These clusters exhibit high sequence similarity to the previously described *cusCBASR* cluster in *K. xylinus* E25 (Ryngajłło et al. 2019a). The *czc2* cluster, similarly as in *K. xylinus* E25, is followed by two genes predicted to encode the CzcS/CzcR two-component system regulating the CzcCBA efflux system in *Pseudomonas aeruginosa* (Perron et al. 2004). Genes in both clusters are significantly downregulated during growth on the glycerol medium, except for *czcS* and *czcR*, which are only mildly repressed (Fig. 7a).

Regarding zinc homeostasis, we found in the *N. hansenii* SI1 genome two genes in consecutive arrangement, which encode homologs of SmtA and SmtB of *Synechococcus elongatus* PCC 7942. SmtA functions as a metallothionein, while SmtB acts as a transcriptional repressor of *smtA*. These proteins are critical for maintaining zinc and other essential ions homeostasis (Huckle et al. 1993). In *Synechococcus elongatus* PCC 7942, SmtA sequesters and detoxifies four Zn^2+^ ions per molecule (Blindauer et al. 2002), while SmtB represses *smtA* expression under low Zn conditions (Huckle et al. 1993). In our analysis, we found that *smtA* and *smtB* were highly expressed in the glycerol medium and are both ranked as top differentially expressed genes (log_2_FC = 3.61, adj. *p-value* = 0 for *smtA* and log_2_FC = 3.41, adj. *p-value* = 0 for *smtB*, Fig. 7b).

Significant induction in expression was observed for the homologs of copper resistance proteins CopA and CopB from *Pseudomonas syringae* pv. *tomato* (Fig. 7c). In *Pseudomonas* species, the cop systems consist of four structural proteins (CopABCD), whose main role is the expulsion of copper from the cell (Hofmann et al. 2021). While *N. hansenii *SI1 lacks homologs of CopC/CopD, *copA1*, *copB*, and an uncharacterised gene (SI1_00647) form a cluster. Phobius predicts SI1_00647 encodes a periplasmic protein, potentially analogous to CopC, though functional validation is needed.

Apart from CopA and CopB proteins, the *N. hansenii *SI1 genome encodes a copper-exporting P-type ATPase homologous to *Staphylococcus aureus* NCTC 8325 CopA (*copA2*), which was significantly upregulated in the glycerol medium (Fig. 7c). Adjacent to *copA2*, we identified a gene encoding a MerP-like mercury scavenger from *Shigella flexneri*, showing mild induction (Fig. 7c).

For cobalt, we observed mild upregulation of genes encoding homologs of cobalt transporter subunits (Online Resource 1: Fig. S6a).

Previously reported TonB-dependent transporters (TBDTs (Ryngajłło et al. 2024)) were predominantly downregulated in the glycerol medium (Fig. 7d and Online Resource 1: Fig. S6b). Notable exceptions included the SI1_00389 gene (significant induction), annotated as colicin I receptor (*cirA* homolog of *E. coli*), and a second *cirA* copy (*cirA*2) with slight upregulation (Fig. 7e).

### Phages and prophages

The results of RAST functional analysis highlighted the “phages, prophages” subcategory as significantly enriched among the downregulated DEGs (Fig. 1b). A total of six genes were assigned to this subcategory, encoding phage packaging machinery, neck, and capsid proteins. Despite being expressed at low levels, these genes exhibited significant downregulation in the glycerol medium cultures (Online Resource 1: Fig. S7).

We further compared these findings with the predictions from the PHASTEST program, which identified two prophage regions in the *N. hansenii* SI1 chromosome. The first region is intact and spans positions 1,089,369–1,117,668 bases, while the second region (designated as questionable) occupies positions 1,990,601–2,010,067 bases. Notably, the genes of the RAST subcategory overlap both prophage loci. By analysing transcription at single-nucleotide resolution, we observed that both regions exhibited predominantly repressed expression in the glycerol medium, with most genes showing statistically significant changes (Online Resource 1: Fig. S8 and Online Resource 2: Table S1).

### Expression of cellulose and acetan synthesis pathways

**Fig. 8:**
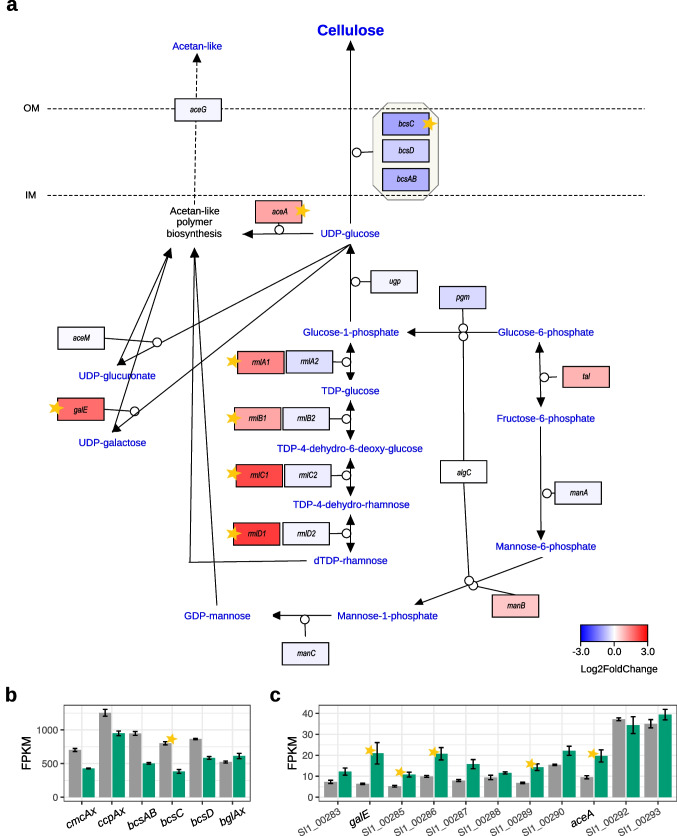
Gene expression changes in the cellulose and acetan-like II biosynthesis pathway in *N. hansenii* SI1. **a** The pathway. The genes are colored according to log_2_ fold change in expression between glycerol and glucose cultures. **b** Expression of the *bcsI* cellulose synthase cluster. **c** Expression of the acetan type II gene cluster. For b–c, transcripts’ mean FPKM values are shown in either gray or green for cells grown in the glucose or glycerol medium, respectively. Bars represent the means from three replicated cultures. Thin black bars denote the standard error. Stars in all panels denote statistically significant changes (called by DESeq2; adjusted *p-value* < 0.01 and |log_2_FC|  ≥ 1)

The cellulose synthesis pathway was generally downregulated in the glycerol medium cultures, with the *bcsC* gene showing significant expression changes (log_2_FC =  − 1.1, adj. *p-value* = 1.76 * 10^−22^; Fig. 8a, b). Similarly, the genes from the other two cellulose synthase (CS) operons (*bcsII* and *bcsIII*) were downregulated (Online Resource 1: Fig. S9a, b).

In contrast, strong and statistically significant induction was observed for the majority of genes involved in acetan-like polymer biosynthesis. Key examples include *aceA*, *galE*, and other genes of the acetan type II cluster (Fig. 8a, c), as well as chromosomal *rml1* genes responsible for dTDP-rhamnose biosynthesis (Fig. 8a and Online Resource 1: Fig. S10c). The genes of the *rml2* cluster, which are located on the p1 plasmid, were mildly downregulated (Fig. 8a and Online Resource 1: Fig. S10d).

## Discussion

Glycerol serves as an important alternative carbon source due to its cost-effective production. Wider exploitation of this polyol for nanocellulose production requires a better understanding of the regulatory mechanisms governing its metabolism in microbial producers like *N. hansenii* SI.

In our prior study (Cielecka et al. 2021b), we compared cellulose yields of *N. hansenii* SI1 on various carbon sources. This strain synthesised cellulose more efficiently on glycerol, producing 3.85 versus 2.29 g/L on glucose. In the study presented here, we aimed to understand the molecular basis of the improved BNC synthesis by analysing transcriptional adaptation to glycerol via RNA sequencing.

The studied transcriptional changes in the central carbohydrate metabolism pathway suggest that when *N. hansenii* SI1 is transferred to the glycerol medium, glycerol uptake and catabolism, gluconeogenesis, PPP, and ED pathway are induced, whereas glycolysis, through the Embden–Meyerhof–Parnas pathway, is repressed (Fig. 2).

Activation of the ED and PPP pathways may be connected to oxidative stress response, since these pathways are important for generating NADPH, which provides reducing power for antioxidant and redox regulatory enzymes, such as the glutathione/glutaredoxin and thioredoxin systems (Holmgren 2001; Grant 2008; Krüger et al. 2011; Chavarría et al. 2013).

We further observed mild induction of the *glp* gene cluster (Fig. 2b), indicating activation of glycerol intake and phosphorylation pathways. Similarly, periplasmic oxidation of glycerol to DHA was likely induced due to the activation of the secondary glycerol dehydrogenase SldAB1 (Fig. 2c). This enzyme’s gene is part of a cluster that also contains genes involved in tagatose and sorbitol metabolism. Such induction may reflect a regulatory adaptation to metabolise alternative carbon sources during glucose deprivation. The combined oxidation of glycerol (in the periplasm) and G3P (cytosolic) likely contributed to oxidative stress generation.

When *N. hansenii* SI1 grows on media where glycerol is the main carbon source, the TCA cycle pathway is generally repressed (Fig. 3). Particularly strong repression was observed for two gene copies encoding malic enzyme isoforms (*maeA1* and *maeA3*; Fig. 3), which catalyse malate degradation to pyruvate and are important for growth on TCA cycle intermediates (Bologna et al. 2007). Additionally, we observed strong repression in the pathway of acetoin biosynthesis from pyruvate (Online Resource 1: Fig. S4). Acetoin (3-hydroxy-2-butanone) is a storage compound secreted under glucose growth conditions to prevent cytosolic and medium over-acidification while storing excess carbon (Xiao and Xu 2007). The significant downregulation of both malate degradation and acetoin biosynthesis suggests they are important regulatory checkpoints for managing energy storage and preventing over-acidification in *N. hansenii* SI1.

Given that glycolysis and TCA cycle progression were repressed, it was not surprising to observe strong downregulation of genes connected to cytoplasmic (NADH-dependent) respiration (Fig. 3c). From this, we infer that the energy status of cells grown in the glycerol medium was lower compared to the glucose medium.

The pathway that was strongly upregulated was that of riboflavin biosynthesis (Fig. 4). This induction likely stemmed from a requirement to replenish flavin cofactors (FMN and FAD), which serve as essential coenzymes for redox enzymes (Olfat et al. 2022; Schnerwitzki and Vabulas 2022).

Surprisingly, we observed significant induction of genes adjacent to the *ribCF* gene that are associated with the methionine salvage pathway (Online Resource 1: Fig. S5). This finding suggests a functional connection between riboflavin and methionine metabolism. Interestingly, such an interaction has been previously documented in *Bacillus subtilis*, where RibR protein regulates FMN riboswitch activity in response to the availability of methionine or taurine in the growth medium (Pedrolli et al. 2015). However, no homolog of the RibR protein was identified in *N. hansenii* SI1, indicating that the regulatory mechanisms linking riboflavin and methionine metabolism in this strain require further investigation.

Another pathway that was strongly induced in the glycerol-grown cultures was the sulphur assimilation pathway (Fig. 5). Interestingly, we previously observed the opposite regulatory trend for this pathway when vitamin C was present in the SH medium (Ryngajłło et al. 2024). At that time, we proposed that strong repression of the sulphur assimilatory pathway served to stabilise redox balance under reduced conditions imposed by vitamin C. If this hypothesis holds true, the observed induction during growth on glycerol suggests that *N. hansenii* SI1 is experiencing oxidative stress under this condition.

Examination of the pathways related to protein degradation and stress response supported the observation that growth in the glycerol medium induces oxidative stress in the cells of *N. hansenii* SI1 (Fig. 6).

The upregulation of genes encoding ATP-dependent proteases and molecular chaperones (Fig. 6a, b) suggests active protein degradation under these conditions.

We observed significant induction of expression of *oxyR1* and *iscR2* genes, which encode transcription factors known to regulate oxidative stress response networks (Fig. 6d; Chiang and Schellhorn 2012). Given that the regulatory functions of these transcription factors are modulated under oxidising conditions, we analysed their regulon expression patterns.

In *E. coli*, OxyR is activated via disulphide bond formation under oxidative stress (Zheng et al. 1998). Its regulon includes ~ 20 genes associated with the adaptive response to redox stress, such as alkyl hydroperoxide reductase (*ahpC* and *ahpF*; Seixas et al. 2022). These genes were significantly upregulated in the glycerol-grown *N. hansenii* SI1 (Fig. 6e), suggesting conservation of this OxyR-mediated response in this strain.

Expression of *iscR* is induced by oxidative stress in *E. coli* (Zheng et al. 2001). IscR regulates genes involved in iron-sulphur ([Fe–S]) cluster biogenesis, including the *iscRSUA* and the *sufABCDSE* operons. Under oxidative stress, the [2Fe–2S] cluster of IscR is oxidised, which causes derepression of the *isc* promoter and induction of the *suf* promoter (Schwartz et al. 2001; Yeo et al. 2006). Our data suggest a conserved regulatory mechanism in *N. hansenii* SI1, as evidenced by the upregulation of *iscA* and the majority of *suf* cluster genes (Fig. 6h).

We observed that the growth of *N. hansenii* SI1 in the glycerol medium induced expression of other genes associated with oxidative stress response, such as *hmp*, which encodes the flavohemoprotein Hmp (Fig. 6e). In *E. coli*, Hmp acts as an oxygen sensor and an amplifier of superoxide stress (Poole et al. 1994; Membrillo-Hernández et al. 1996). Our findings imply that the induction of *hmp* may represent an important regulatory node during oxidative stress in *N. hansenii* SI1. Conversely, *hmp* expression was significantly downregulated under reducing conditions (Ryngajłło et al. 2024).

Another gene significantly upregulated in our study encodes the bifunctional PutA protein, which catalyzes oxidation of proline to glutamate (Fig. 6e). In Gram-negative bacteria, PutA has been shown to possess both FAD-dependent proline dehydrogenase and NAD^+^-dependent glutamate semialdehyde dehydrogenase activities (Liu et al. 2017). Its role in enabling proline utilisation as a carbon/nitrogen source during nutrient scarcity is well documented (Tanner 2008). Sequence analysis of PutA in *N. hansenii* SI1 revealed the absence of an N-terminal RHH domain, suggesting it may lack transcriptional regulatory functions reported for *E. coli* PutA (Larson et al. 2006). Notably, enteric bacteria exhibit catabolite repression of *putA*, with glucose suppressing its expression (Chen and Maloy 1991). The upregulation of *putA* in *N. hansenii* SI1 grown in the medium where glucose is replaced with glycerol implies potential conservation of this regulatory mechanism. However, further studies are required to confirm these observations.

Genes encoding methionine sulphoxide reductases represent another group of repair proteins that were induced in the cultures grown in the glycerol medium (Fig. 6g). We found particularly strong induction of the gene encoding a homolog of the *E. coli* MsrB protein, previously demonstrated to efficiently reduce protein-bound methionine sulphoxide residues (Grimaud et al. 2001). The same study revealed that *msrB* is important for resistance to cadmium, which induces oxidative stress.

Methionine is highly sensitive to oxidation, which can cause structural modifications that alter or inhibit protein function (Vogt 1995). Consequently, diverse protective mechanisms have evolved in living organisms to control oxidised methionine levels. Our findings align with this concept, since we show that under oxidative stress, *N. hansenii* SI1 may activate two routes: one that recycles methionine through the salvage pathway (Online Resource 1: Fig. S5a) and another that induces genes encoding repair enzymes for oxidised methionine, such as the sulphoxide reductase *msrB* (Fig. 6g).

Metal ions are essential cofactors for numerous biological processes in all living organisms. However, metal overaccumulation can be toxic, as it may generate reactive oxygen species (ROS) via Fenton chemistry (Chiang and Schellhorn 2012). Consequently, tight regulation of intracellular metal ion concentrations of metal ions is critical. Our transcriptomic analysis highlighted significant changes in the expression of genes connected to the regulation of homeostasis of various metal ions.

By analysing the gene expression profiles, we found that the cobalt-zinc-cadmium resistance system, represented by the *czcCBA* clusters, was significantly repressed in the culture grown on the glycerol medium (Fig. 7). Conversely, genes involved in zinc ion homeostasis—*smtA*, encoding metallothionein, and its repressor *smtB*—were expressed at levels exceeding three-fold higher compared to controls (Fig. 7b). This suggests that the elevated Zn^2+^ concentrations observed in glycerol-grown cultures may stem from protein degradation, as we highlighted above. Our results show that under these conditions, zinc ions are preferentially sequestered by SmtA metallothionein rather than being actively exported by the czcCBA efflux system.

Our transcriptomic results further revealed activation of copper homeostasis in the cultures grown in the glycerol medium, as evidenced by significant upregulation of genes encoding copper expulsion systems (Fig. 7c). Additionally, mild induction was observed for the predicted mercury scavenger *merP* (Fig. 7c) and cobalt transporter subunits (*cbtA1*, *cbtA2*, *cbtB*; Online Resource 1: Fig. S6a).

Because we observed that the majority of genes encoding TonB-dependent transporters were significantly downregulated in the glycerol medium (Fig. 7d and Online Resource 1: Fig. S6b), we inferred that iron acquisition mechanisms were repressed under these conditions. One exception among genes in this category was the significant upregulation of a homolog of the colicin I receptor of *E. coli* encoded by the *cirA1* gene (Fig. 7e). In *E. coli*, the *cir* gene product may function as a receptor for an unidentified siderophore and is regulated by the carbon catabolite repression system (Griggs et al. 1990). The induction of *cirA1* in a glucose-limited medium suggests similar regulatory mechanisms in *N. hansenii* SI1. However, further studies are required to elucidate its functional role in glycerol-grown cultures.

We further observed strong downregulation of genes encoding phage proteins across two distinct prophage regions integrated within the chromosome (Online Resource 1: Figs. S7 and S8). These findings suggest that suppression of phage expression may mitigate oxidative stress and promote genome stability by reducing potentially damaging phage activity. However, the mechanism underlying this potential regulatory link warrants experimental validation.

Finally, we examined the expression of the cellulose biosynthesis pathway (Fig. 8). The main (*bcsI*, Fig. 8b) and auxiliary (*bcsII*, *bcsIII*, Online Resource 1: Fig. S9a, b) cellulose synthase operons exhibited strong downregulation in the glycerol medium. Despite this transcriptional repression of *bcs* operons, an increased BNC synthesis was observed in the glycerol medium (Cielecka et al. 2021b). This raises the question: Why does *N. hansenii* SI1 exhibit enhanced BNC production in the glycerol medium?

Firstly, a higher yield of BNC in the glycerol medium may be attributed to the altered and more efficient glycerol metabolism compared to glucose, as previously reported (Jung et al. 2010; Wang et al. 2018). Through metabolic flux analysis of central carbohydrate metabolism in *K. xylinus* CGMCC 2955 (*Gluconacetobacter* in the original work), Zhong and co-authors found that ca. 47.96% of glycerol was transformed into BNC, compared to only 19.05% of glucose (Zhong et al. 2013). These observations align with gene expression changes in central carbohydrate metabolic pathways observed in this study, as we discussed above (Fig. 2).

Secondly, pH also has to be considered, as it is an important factor for cellulose production. A decline in pH during growth on glucose is detrimental to BNC biosynthesis (Çoban 2011; Cielecka et al. 2020). Importantly, cultivation at a pH below 3.5 halts cellulose synthesis and impairs bacterial growth (Embuscado et al. 1994). Zhong and co-authors calculated that 40.03% of glucose is converted to gluconic acid, leading to a sharp pH reduction—a phenomenon absent in glycerol cultures (Zhong et al. 2013). Consistent with this, our previous work showed that the final pH of *N. hansenii* SI1 grown on glycerol remained near 6.0 (Cielecka et al. 2021b). The transcriptomic profiles obtained in this study further support a higher pH environment in the glycerol cultures, since we observed that the genes connected to organic acid synthesis or metabolism, as well as those preventing over-acidification, were strongly downregulated in comparison with the glucose culture (Fig. 3 and Online Resource 1: Fig. S4).

Another explanation for the elevated BNC synthesis in the glycerol culture (not directly observed here) could involve posttranslational activation of cellulose synthase. Allosteric activation of the catalytic subunit BcsA by c-di-GMP (bis-(3′,5′)-cyclic di-guanosine-mono-phosphate) occurs via binding to its PilZ domain (Ross et al. 1985; Römling 2002; Hengge et al. 2019). Intracellular c-di-GMP levels are dynamically regulated by proteins with opposite enzymatic activities: diguanylate cyclases (DGCs) and c-di-GMP-specific phosphodiesterases (PDEs), which catalyze c-di-GMP formation and degradation, respectively (Tal et al. 1998; Römling 2012). It has been shown by Qi and co-authors that DGC2 (*Ax*DGC2) harbours a flavin (FAD) co-factor-binding Per-Arnt-Sim (PAS) domain and regulates c-di-GMP levels in response to fluctuations in cellular redox status or oxygen concentration (Qi et al. 2009). These authors have shown that the catalytic activity of the GGDEF domain of DGC2 depends on the redox status of the FAD cofactor, with the oxidised form resulting in higher catalytic activity and stronger substrate inhibition. On the other hand, it has been reported that the PAS domain of PDEA1 contains heme and is regulated by reversible binding of O_2_ (Chang et al. 2001). In this case, oxygen suppresses the activity of PDEA1. Our transcriptomic results suggest that glycerol metabolism induces oxidation of the cytosol. Under these conditions, FAD and heme cofactors likely remain oxidised, leading to activation of DGC2 and deactivation of PDEA1. This shift would elevate c-di-GMP concentrations, enhancing BcsA activation and promoting BNC synthesis.

Finally, we reported significant induction in the expression of genes associated with acetan-like polymer biosynthesis in the glycerol cultures (Fig. 8a, c). Conversely, these genes were strongly downregulated in *N. hansenii* SI1 cultivated in the SH medium supplemented with vitamin C, where lower BNC yields were recorded compared to the unmodified SH medium (Cielecka et al. 2021b). An increased concentration of acetan likely stimulates BNC production, as demonstrated in *K. xylinus* BPR2001 (formerly *Acetobacter xylinum*), where this EPS enhances culture viscosity, promotes BNC/cell dispersion, and improves oxygen or nutrient uptake efficiency (Ishida et al. 2002). The transcriptomic findings of this study align with previously reported observations.

## Supplementary Information

Below is the link to the electronic supplementary material.

ESM 1 (PDF 751 KB)

ESM 2 (XLSX 839 KB)

## Data Availability

Sequence data (raw NGS reads) that support the findings of this study have been deposited at the NCBI database under the BioProject accession: PRJNA821681 and BioSample accessions: SAMN42361398-SAMN42361400. Other data is provided within the supplementary information files.
