# Supplementary material for: Transcriptional reprogramming of Novacetimonas hansenii SI1 during growth on glycerol
Source: Appl Microbiol Biotechnol. 2025 Sep 2;109(1):194. doi: 10.1007/s00253-025-13583-2 (PMC12405394; doi:10.1007/s00253-025-13583-2)
Supplement: Supplementary file 1 — (PDF 751 KB) [file 253_2025_13583_MOESM1_ESM.pdf]

Applied Microbiology and Biotechnology

Transcriptional reprogramming of *Novacetimonas hansenii* SI1 during growth on glycerol

Małgorzata Wlaźlak[1*], Izabela Cielecka[1], Maurycy Daroch[2]

[1]Institute of Molecular and Industrial Biotechnology, Lodz University of Technology, B. Stefanowskiego 2/22, 90-537 Lodz, Poland

[2] School of Environment and Energy, Peking University Shenzhen Graduate School, Shenzhen, China.

*Corresponding author

Email: malgorzata.ryngajllo@p.lodz.pl

Telephone: +48 0426313354

ORCID: 0000-0002-0430-0102

Supplementary file 1: Supplementary Figures

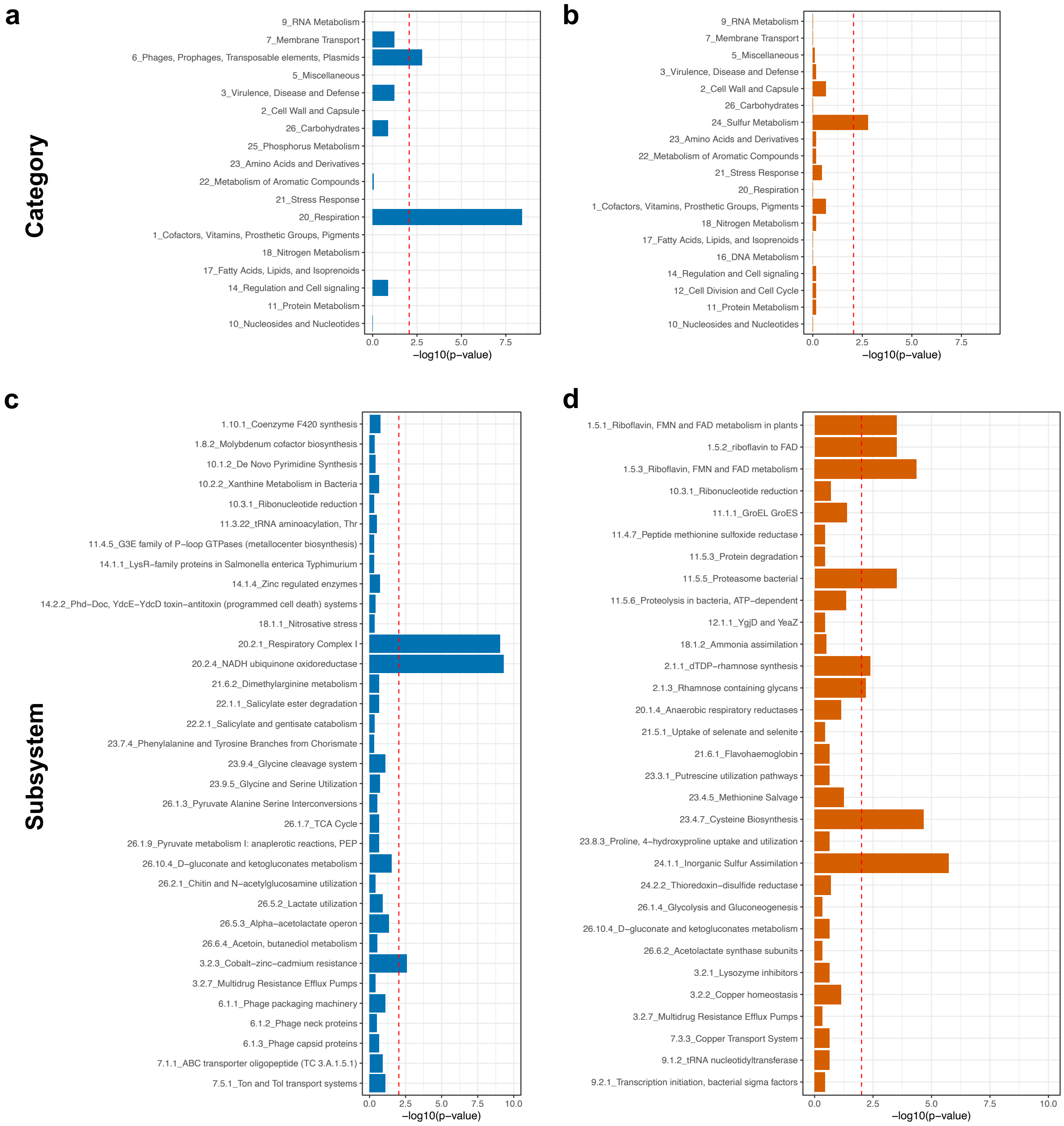

Fig. S1 Results of functional enrichment analysis based on RAST annotation of *N. hansenii* SI1 genome. Displayed are enriched RAST: categories (**a-b**), subsystems (**c-d**). The enriched functional bins of the up-regulated and the down-regulated DEGs are displayed as blue and orange bars, respectively. Shown are the results of the one-sided Fisher's Exact test (one-tailed). In case of the subsystems, for clarity, presented are only the bins, which scored the adjusted *p-value* below 0.5. The red, dashed vertical line indicates the adjusted *p-value* threshold of 0.01

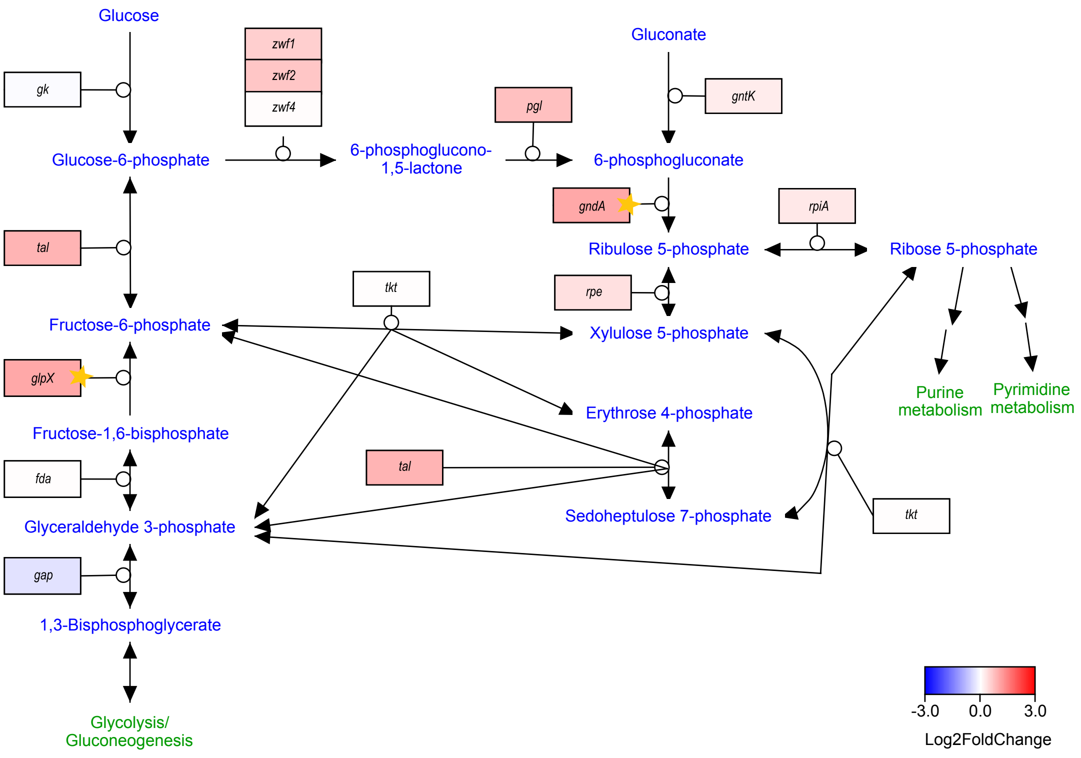

Fig. S2 Trancriptomic changes in the Pentose phosphate pathway in *N. hansenii* SI1. Genes are colored according to $\log_2$ fold change in expression between glycerol and glucose cultures. Stars denote statistically significant changes (called by DESeq2; adjusted *p-value* < 0.01 and $|\log_2 FC| \geq 1$)

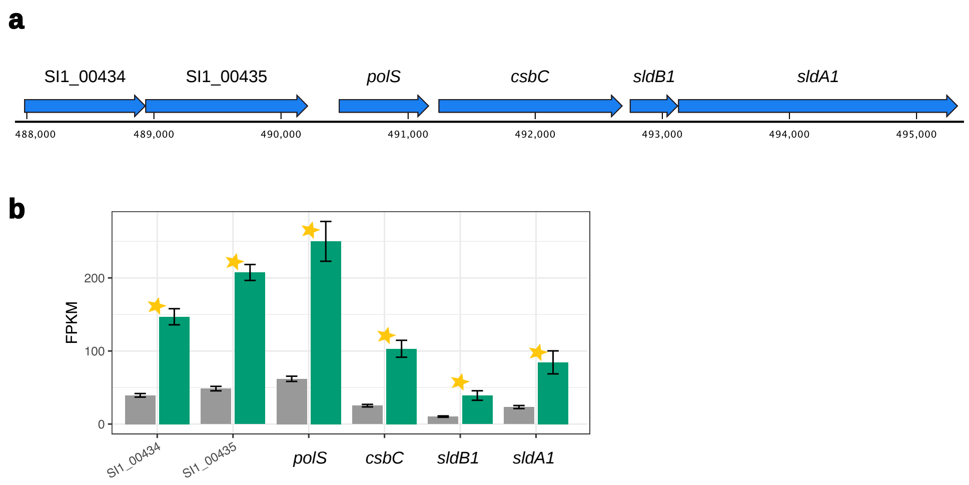

Fig. S3 Genomic context and expression changes of genes located in close proximity to genes encoding glycerol dehydrogenase subunits (*sldA1* and *sldB2*). **a** The structure of genes of the cluster in the genome of *N. hansenii* SI1. **b** Expression of genes of the cluster. The gene codes and symbols refer to the following predicted functions: SI1_00434 – tagatose kinase; SI1_00435 – tagatose 6-phosphate 4-epimerase; *polS* (SI1_00436) – sorbitol dehydrogenase, *csbC* (SI1_00437) – putative metabolite transport protein; *sldB1* (SI1_00438) – glycerol dehydrogenase, small subunit; *sldA1* (SI1_00439) – glycerol dehydrogenase, large subunit. Transcripts mean FPKM values are shown in gray or green for cells grown in either the glucose or the glycerol medium, respectively. Bars represent the means from 3 replicated cultures. Thin black bars denote standard error. Stars denote statistically significant changes (called by DESeq2; adjusted *p-value* < 0.01 and $|\log_2 FC| \geq 1$)

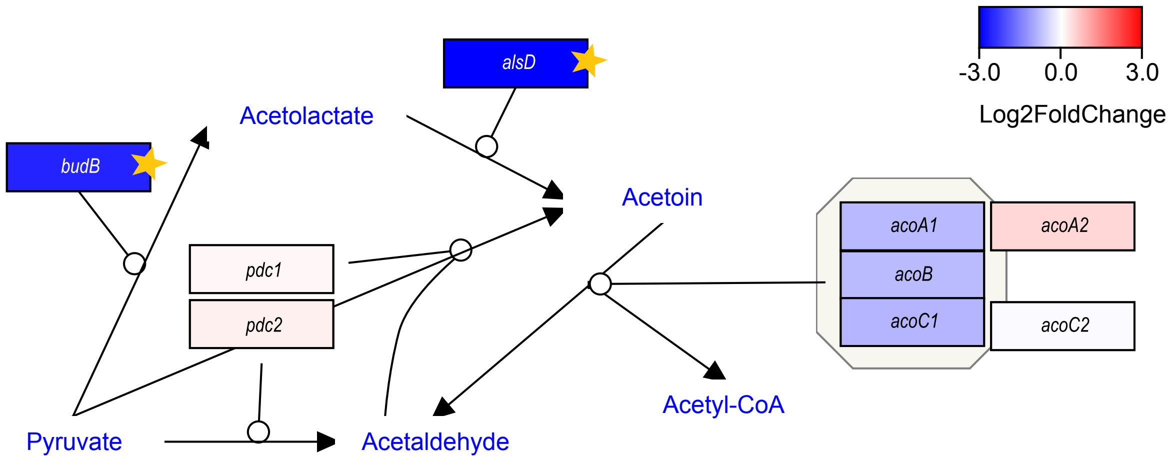

Fig. S4 Trancriptomic changes in the putative acetoin metabolism pathway in *N. hansenii* SI1. Genes are colored according to $\log_2$ fold change in expression between glycerol and glucose cultures. Stars denote statistically significant changes (called by DESeq2; adjusted *p-value* < 0.01 and $|\log_2 FC| \geq 1$)

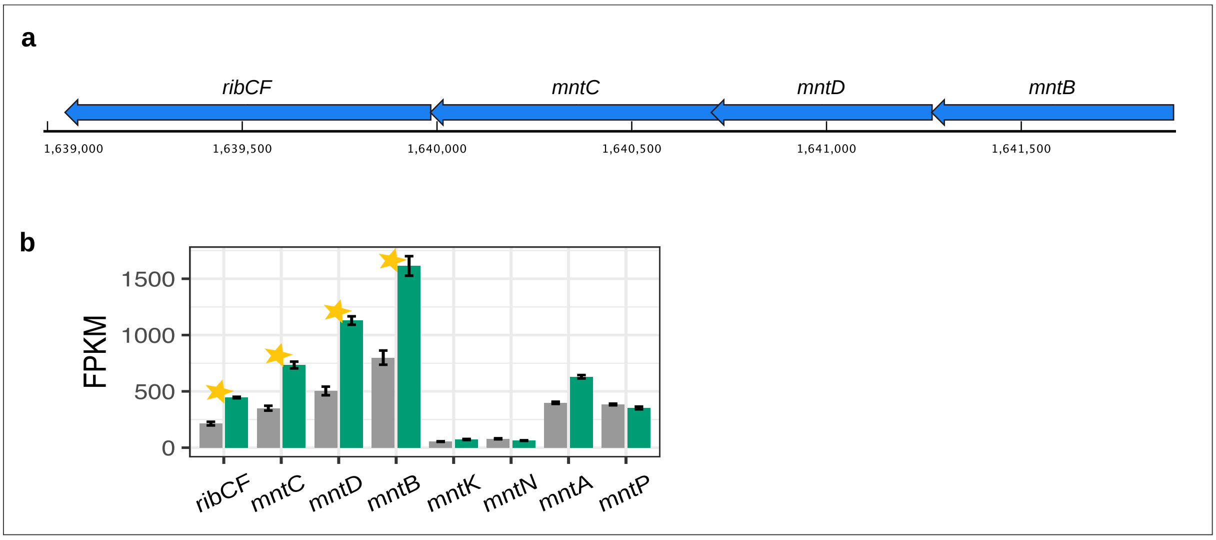

Fig. S5 Structure and trancriptomic changes of gene loci enclosing *ribCF* and methionine salvage pathway genes. **a** Structure of the loci. **b** Expression of the predicted methionine salvage pathway genes located next to *ribCF* (*mntC*, *mntD*, *mntB*)and other genomic loci (*mntK*, *mntN*, *mntA*, *mntP*). Transcripts mean FPKM values are shown in gray or green for cells grown in either the glucose or the glycerol medium, respectively. Bars represent the means from 3 replicated cultures. Thin black bars denote standard error. Stars denote statistically significant changes (called by DESeq2; adjusted *p-value* < 0.01 and $|\log_2 FC| \geq 1$)

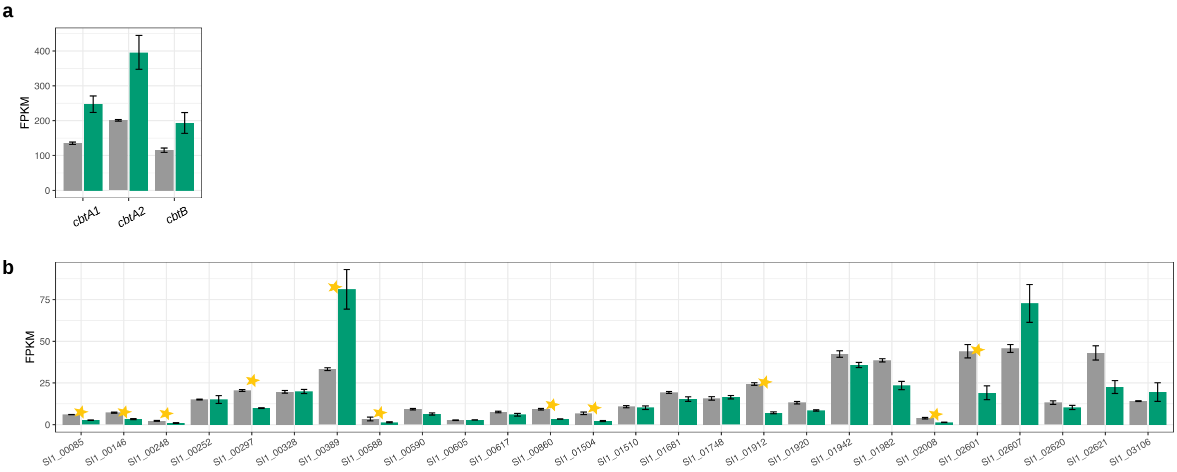

Fig. S6 Expression of genes involved in cobalt and iron homeostasis. **a** Genes encoding cobalt transporters. **b** Low expressed TonB-dependent receptor genes. Transcripts mean FPKM values are shown either gray or green for cells grown in either the glucose or the glycerol medium, respectively. Bars represent the means from 3 replicated cultures. Thin black bars denote standard error. Stars denote statistically significant changes (called by DESeq2; adjusted *p-value* < 0.01 and $|\log_2 FC| \geq 1$)

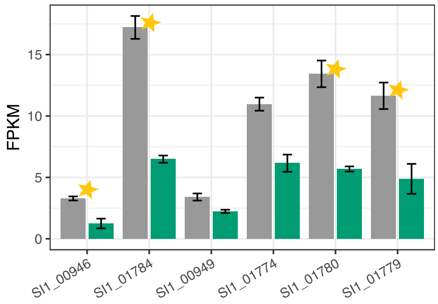

Fig. S7 Expression of the genes encoding phage proteins as annotated by the "Phages, Prophages" RAST subcategory. Transcripts mean FPKM values are shown either gray or green for cells grown in either the glucose or the glycerol medium, respectively. Bars represent the means from 3 replicated cultures. Thin black bars denote standard error. Stars denote statistically significant changes (called by DESeq2; adjusted *p-value* < 0.01 and $|\log_2 FC| \geq 1$)

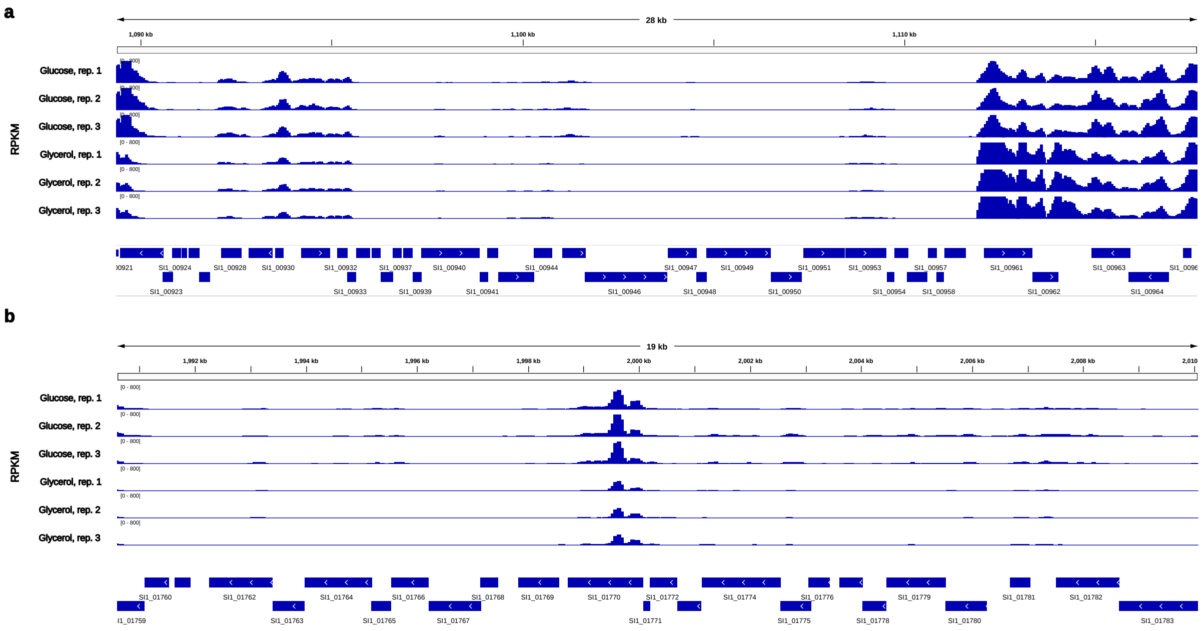

Fig. S8 Sequence coverage plots for samples from cultures grown on glucose or glycerol as the main C source in the two prophage loci predicted by PHASTEST program. **a** First region of 28.3 kbp (1,089,369-1,117,668). **b** Second region of 19.4 kbp (1,990,601-2,010,067). Read counts from each library were normalized using RPKM

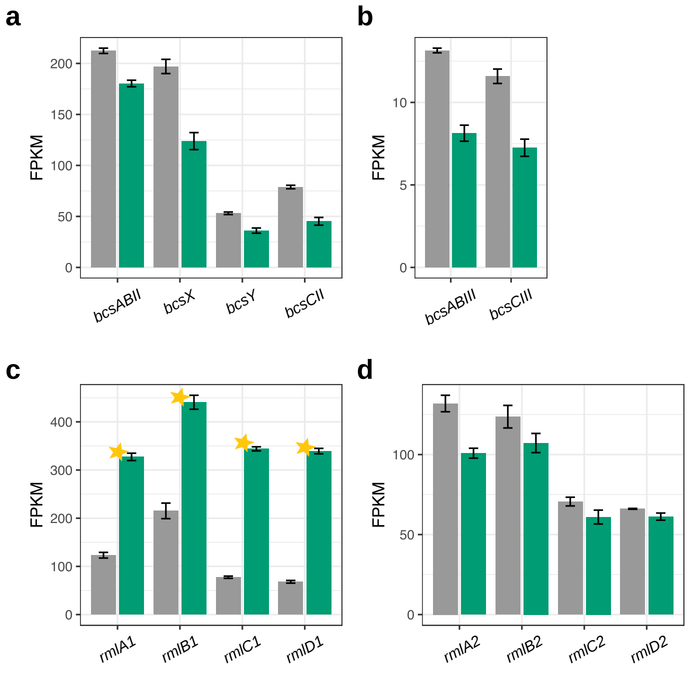

Fig. S9 Expression of cellulose synthase and acetan type II clusters. **a** Expression of *bcs*II cluster **b** Expression of *bcsII* cluster **c** Expression of the *rml1* cluster of the chromosome **d** Expression of *rml2* cluster of the p1 plasmid. Transcripts mean FPKM values are shown either gray or green for cells grown in either the glucose or the glycerol medium, respectively. Bars represent the means from 3 replicated cultures. Thin black bars denote standard error. Stars denote statistically significant changes (called by DESeq2; adjusted *p-value* < 0.01 and $|log_2FC| \geq 1$)
